# Effect of Subliminal Lexical Priming on the Subjective Perception of Images: A Machine Learning Approach

**DOI:** 10.1371/journal.pone.0148332

**Published:** 2016-02-11

**Authors:** Dhanya Menoth Mohan, Parmod Kumar, Faisal Mahmood, Kian Foong Wong, Abhishek Agrawal, Mohamed Elgendi, Rohit Shukla, Natania Ang, April Ching, Justin Dauwels, Alice H. D. Chan

**Affiliations:** 1 School of Electrical and Electronic Engineering, Nanyang Technological University, Singapore, Singapore; 2 INRIA Sophia Antipolis, Sophia Antipolis, France; 3 Okinawa Institute of Science and Technology, Okinawa, Japan; 4 Center for Cognitive Neuroscience, Duke-NUS Graduate Medical School, Singapore, Singapore; 5 University of British Columbia, Vancouver, Canada; 6 University of Wisconsin-Madison, Madison, United States of America; 7 Linguistics and Multilingual Studies, School of Humanities and Social Sciences, Nanyang Technological University, Singapore, Singapore; Centre de Neuroscience Cognitive, FRANCE

## Abstract

The purpose of the study is to examine the effect of subliminal priming in terms of the perception of images influenced by words with positive, negative, and neutral emotional content, through electroencephalograms (EEGs). Participants were instructed to rate how much they like the stimuli images, on a 7-point Likert scale, after being subliminally exposed to masked lexical prime words that exhibit positive, negative, and neutral connotations with respect to the images. Simultaneously, the EEGs were recorded. Statistical tests such as repeated measures ANOVAs and two-tailed paired-samples t-tests were performed to measure significant differences in the likability ratings among the three prime affect types; the results showed a strong shift in the likeness judgment for the images in the positively primed condition compared to the other two. The acquired EEGs were examined to assess the difference in brain activity associated with the three different conditions. The consistent results obtained confirmed the overall priming effect on participants’ explicit ratings. In addition, machine learning algorithms such as support vector machines (SVMs), and AdaBoost classifiers were applied to infer the prime affect type from the ERPs. The highest classification rates of 95.0% and 70.0% obtained respectively for average-trial binary classifier and average-trial multi-class further emphasize that the ERPs encode information about the different kinds of primes.

## 1 Introduction

Understanding how affective content influences decision making without being consciously perceived has been an area of active research [1]. Such evaluative and affective responses rely on the interplay of underlying emotional and cognitive processes, which are assumed to be instantaneous and automatic [2, 3]. When a subliminal presentation of a prime object prejudices or changes one’s evaluation of a subsequently presented target object in the direction of the affective valence of the prime object, there is said to be an affective priming effect [4]. This effect occurs faster and more accurately when the prime and target are affectively congruent (i.e., positive-positive or negative-negative) than when affectively incongruent (i.e., positive-negative or negative-positive) [4, 5].

Various past and recent studies have found the affect priming effect across an array of prime stimuli, from emotional pictures [6–11] to emotional words [12–15] or both [16–18]. Most of these studies elicited the use of ERPs alongside behavioral performance measurements and, as a result, have identified two emotion-related ERP components [19]. The early posterior negativity (EPN) occurs around 100–200 ms [20, 21] and the late positive complex (LPC) at around 200–500 ms [22, 23]. Neurophysiological analysis of subliminal priming has made the tracking of these seemingly automatic unconscious processes visible, allowing an online measurement with temporal resolution and complex information processing efficiently, otherwise not possible with standard behavioral measurements alone, such as tracking reaction time or accuracy scores [17, 18, 24]. ERP studies have also been used in applications such as object recognition [25], decoding of visual attention [26, 27], and prediction of human cognitive states [28].

However, while utilizing EEG has been beneficial in investigating brain-behavior relationships, can ERP data accurately and efficiently reveal one’s intention or what one is thinking? Is it possible to ‘decode’ an individual’s thoughts or even unconscious mental state based only on measurements of their brain activity? It seems that this prospect has remained purely hypothetical [29]. Therefore, in this present study, we address the question of whether unconscious mental states can be consistently decoded from performance patterns during a subliminal affective priming task. Standard ERP analyzes and pattern classification techniques, i.e., Support Vector Machine (SVM) and AdaBoost classifiers, are implemented in a comparative study to provide additional evidence to bolster the reliability of ERP data alongside behavioral data. Pattern classifiers facilitate the integration of neural activity into a decision variable so as to compute the comparison of performance parameters with corresponding behavioral performance. Classifiers such as SVM and AdaBoost have been found to perform extremely well for brain data [30–33]. The main goal of the present study is to quantitatively assess the ability of ERP metrics to successfully predict the affective valence (positive, negative, and neutral) of the visual lexical stimulus (prime word) presented to the participant and hence reconstruct the mental states across observers according to ERP data.

To the best of our knowledge, no study published to date has used pattern classifiers like the SVM in characterizing the neural correlates of behavioral performance during a subliminal priming presentation of affective cross-domain stimuli so as to predict the mental states of young, healthy participants. The primes used in the present study are words across three valences (positive, negative, and neutral), and subsequent target stimuli are originally neutral images (cf. Gibbons, 2009 for similar experimental stimuli). In a recent study by Grotegerd et al. (2013), SVM was used in a subliminal priming experiment on unipolar and bipolar depression patients but with emotional faces as both prime and target stimuli. Philiastides et al. (2006), Philiastides and Sajda (2006), and Das et al. (2010) used pattern classifiers during perceptual decision paradigms (single-trial EEG) for predicting perceptual decision biases, and both of the prime-target stimuli were pictures (face/car paradigm). Bode et al. (2012) likewise used SVM in their multivariate pattern analyzes to study choice priming biases in a perceptual decision paradigm with static noise-masked images of pianos and chairs.

The use of subliminal emotional stimuli (words and images) in our experimental design has various implications on the predictions for this study. We expect that the affective primes will cause participants to respond differently to the target images and emotional-content-dependent ERP modulations can be observed as early as at the P1 (40–120 ms), N1 (80–170 ms) and P2 (100–210 ms) time windows, characteristic of the EPN [34]. In addition, the LPC can be expected in the posterior regions due to a shift in likeness judgment in the positively primed condition not found in the negative and neutral conditions [35–37]. Specifically, the affective word priming conditions would elicit a lexical priming effect, notably the P300 and N400 effects, associated with attentional capture, evaluation, and memory encoding [38–40]. There would also be a more pronounced late ERP component in the posterior regions of the right hemisphere rather than the left, as the right hemisphere is said to play a dominant role in emotional prosody and semantics [41]. Lastly, the SVM as a pattern classifier is predicted to be a successful tool for discriminating among the prime types and thereby the mental states of participants.

The preliminary results obtained were previously published in [42].

## 2 Methods

In this section, we describe the experimental protocol, stimuli selection, procedure, EEG signal recording, preprocessing, feature extraction, and classification techniques. The theoretical background of SVM and AdaBoost are also briefly reviewed.

### 2.1 Participants

Forty English-speaking students (26 males, 14 females; M = 22.3 years, ranging 19–33) at Nanyang Technological University volunteered to participate in this study. All had normal or corrected-to-normal vision and were naive to the purpose of the experiment. The Edinburgh Handedness Inventory [43] was administered to determine handedness (39 right-handed and 1 left-handed). The Nanyang Technological University Institutional Review Board approved this study and experimental paradigm. All participants gave informed written consent and received monetary remuneration for their participation.

### 2.2 Stimuli

An initial pilot study was conducted to construct the stimulus set. Six hundred and seventy-five images were acquired at random from the Internet and converted to grayscale. All images depicted objects or places that could be named with a single word (e.g., bangles, restaurant). Each image in the initial set was paired with a positive, negative, and neutral word prime based on suggestions from four analysts of varied cultural backgrounds. Words that were semantically unrelated to the image were considered neutral word primes. The resultant word-image pairs were then submitted to a preliminary rating study to determine the strength of association between each image and its three suggested words.

In the rating study, 10 participants (6 females) rated the following on a 7-point Likert scale:

1. the likability of the depicted object/place in the image (least = 1; most = 7),
2. the ease of recognition of the depicted object/place in the image (difficult = 1; easy = 7),
3. the strength of association between the image and each of its three prime words (no association = 1; high association = 7) and,
4. the affect valance of that association (very negative = 1; neutral = 4; very positive = 7).

The order of the images and the three prime words to be rated per image was randomized among participants. Each image and its word primes were rated by all 10 participants. The selection of suitable word-image pairs for each affect type was based on the following criteria: positive word-image pairs were rated between 5 and 7 for affect valence by at least 80% of participants, neutral pairs were rated 4 by at least 80% of participants, and negative pairs were rated between 1 and 3 by at least 80% of participants. This procedure created a stimulus pool of 417 word-image pairs consisting of 163 positive pairs, 128 neutral pairs, and 126 negative pairs. No images passed the scoring criteria for more than one affect type, thus there are no repeated images within the stimulus pool.

Next, 150 word-image pair consisting of 50 positive pairs, 50 neutral pairs, and 50 negative pairs were selected from the stimulus pool for the experiment. To ensure that the images within each condition had similar distributions of qualities, rating scores for each image-word pairs were averaged across the participants and submitted to a separate one-way ANOVA for verification. Affect valance scores were highly significant between all three affect conditions (F(2,147) = 383.68, p<0.001). The mean association valance scores for the positive, negative, and neutral conditions were 5.40, 2.77, and 3.96, respectively. Likability (Likert score) and ease of recognition scores were non-significant between the three conditions (F(2,147) = 0.57, p>0.1; F(2,147) = 0.15, p>0.1).

**Fig 1:**
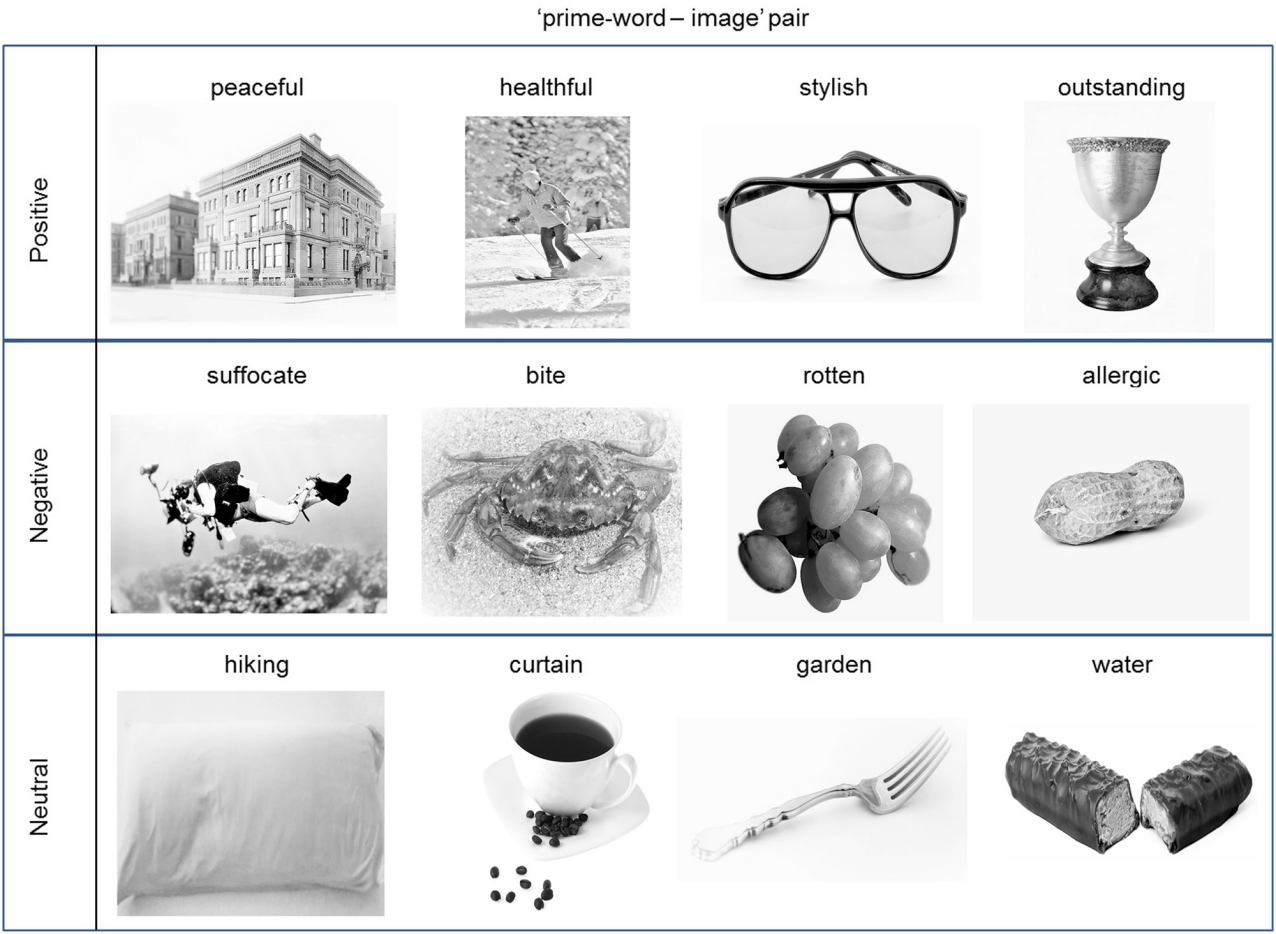
Three types of ‘prime-word—image’ pairs.

It should be noted that the association strength for positive and negative conditions was significantly different (F(1,98) = 45.30, p<0.001). To limit this effect, only word-image pairs with mean association strengths greater than 3 were retained as stimuli. The mean association strength scores for positive, negative, and neutral conditions were 5.14, 3.70, and 1.56, respectively. A few samples of prime word—image pairs chosen are shown in Fig 1.

Words were used as subliminal affective primes for the images. Visual stimuli were presented on the LCD monitor (Dell computer, resolution 800 × 600, refresh rate of 60 Hz, color depth of 16-bit) at a viewing distance of 60 cm.

### 2.3 Experiment Procedure

**Fig 2:**
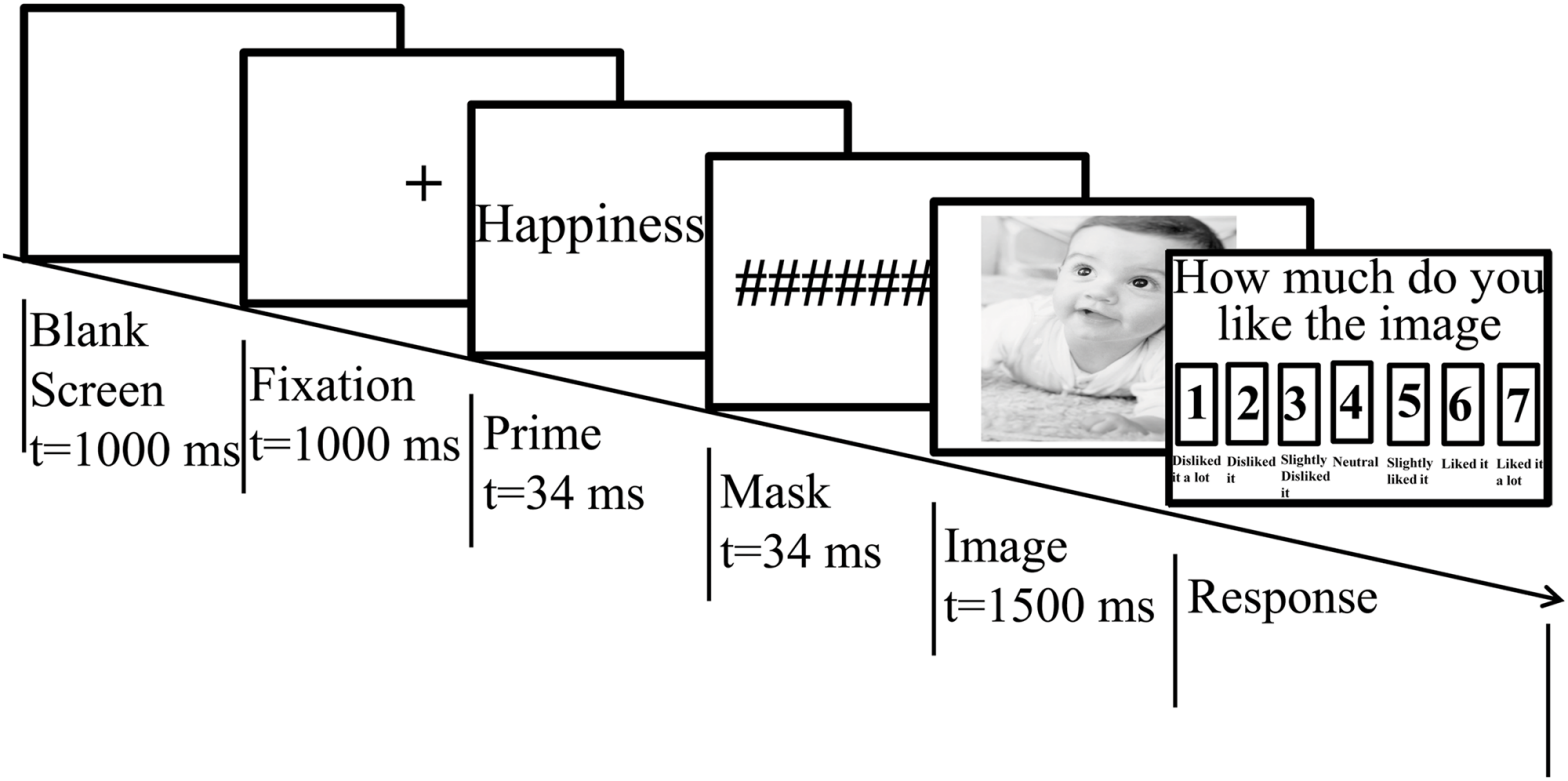
Experimental sequence for a single-trial consisting of a blank screen, fixation mark, prime stimulus, mask, main stimulus, and response box.

The sequence of the events in a single-trial is schematized in Fig 2. The start of each trial was triggered by presenting a blank screen for 1000 ms followed by displaying a fixation point, the mark ‘+’ at the center of the white screen for 1000 ms. Offset from the fixation point, a prime word was presented subliminally for 34 ms, followed by a mask ‘##########’ for 34 ms. The duration of the prime words was carefully chosen according to the previous literature showing that a presentation of a simple shape [44], or a word [45, 46] for 34 ms causes a subliminal priming effect. Following the mask, a target image was exposed for 1500 ms. On the target image offset, participants were prompted to rate how much they liked the presented image on a 7-point Likert scale, ranging from one (liked the least) to seven (liked the most). The prompt remained in view until the participant’s response was obtained, which was made by pressing one of the seven buttons of the keyboard. Simultaneously, the EEG signals were recorded. The inter-trial interval was fixed at 1000 ms. Each participant performed 150 trials of the rating task, split up into 5 different blocks consisting of 30 trials per block, with a short break between the blocks. The sequence of ‘prime word—image’ pairs was randomized between blocks, and each pair was unique.

An additional procedure was carried out with 10 participants (8 males; mean age of 23.4), different from the participants in the experiment with primes, to determine image rating behavior in the absence of subliminal priming. The procedure for the experiment without primes excluded the 34 ms-long prime word presentation from the original sequence and was otherwise identical.

### 2.4 EEG Recording and Preprocessing

The EEG was recorded using a 32-channel HydroCel GSN (HCGSN) sensor array from Electrical Geodesic Inc. (EGI), and arranged according to the 10–20 system [47] at a sampling rate of 250 Hz. Net Amplifier 300 was used to amplify the signal at each electrode by a factor of approximately 20. The EEG data were processed with EEGLab [48] running in the MATLAB (Mathworks, Natick, MA, USA) environment. The recorded data were band pass filtered at 0.1–30 Hz and then referenced to the average of all electrodes.

Epochs for ERPs were collected at −1000 ms to 1500 ms around the image onset for each priming condition. The baseline was set to be −1000 ms to 0 ms. Infomax [49], an independent components analysis algorithm implemented in EEGLAB, was applied to the remaining data to eliminate eye, muscle, and line noise artifacts. In a small number of participants, noisy channels in raw data were removed and interpolated after back-projection using spherical spline interpolation. Individual epochs were then visually inspected for the remaining artifacts, and 8.2% of all epochs were rejected from the final analysis.

### 2.5 ERP Feature Extraction

We extracted features from the time, frequency, and time-frequency domains, which include window-based mean amplitudes, relative power from alpha, beta, and gamma bands, power spectral density (PSD) estimates from short time Fourier transform (STFT), and wavelet coefficients from the discrete wavelet transform (DWT).

The pre-processed artifact-free single-trial ERP waveforms were averaged across the trials for each participant, electrode, and prime affect type. The mean amplitudes in 25 ms discrete time windows, from 0 to 500 ms of the ERP segment, after the image onset, were then extracted. The neural activity associated with different prime affect conditions, the variation as time elapses, and the existence of ERP components related to various brain activities could be measured and differentiated among the prime affect types. The mean amplitudes are used as input features to the classifiers.

Further, we applied fast Fourier transform (FFT) to the single-trial ERPs, computed the power spectrum, and extracted relative power corresponds to alpha (8–12 Hz), beta (13–30 Hz), and gamma (30–60 Hz) bands. The extracted values are then fed to the classifiers.

For a non-stationary signal like ERP, the time-frequency analyzes such as STFT and wavelet transform (WT) help identifying the time varying spectral content. STFT is applied to single-trial ERPs with a Hamming window of 128 point length with 50% overlap. Then, the FFT algorithm is applied to each segment. The PSD estimates of each segment, corresponding to different frequency bands, are extracted and used as input to the classifier.

In the STFT algorithm, a fixed duration time window is applied across all frequencies. In general, high-frequency signals require shorter time-windows and low-frequency signals require longer time-windows to optimally characterize the signal. This limitation is eliminated by using WT, in which the window size varies across the frequencies.

The DWT is used to calculate the wavelet coefficients at discrete intervals of time and scale. This technique provides optimal resolution in both the time and the frequency domains. The DWT of a signal *x*(*t*) is expressed as:

$$\begin{array}{c} DWT\left(j,k\right)=\frac{1}{\sqrt{2^{j}}}\int_{-\infty }^{\infty }x\left(t\right)\psi \left[\frac{t-2^{j}k}{2^{j}}\right]dt, \end{array}$$ (1)

where *a* and *b* are replaced by 2*^j^* and 2*^j^k* respectively.

We applied decomposition levels up to 5 to the single-trial ERP data. The approximate coefficients (*cAj*) at level *j* were used for reconstructing the signal. We observed that the significant ERP features were kept well preserved up to the decomposition level-3. Hence, we selected level-3 decomposition for further analyzes. Tests were conducted with several mother wavelet functions such as Daubechies (db2, db4, and db8), Symlet (sym8), and Biorthogonal (Bior4.4) waves, and the one that yielded the maximum efficiency was selected for the application [50–52]. The approximate coefficients at level-3 (*cA*3) were used for classification.

The above-mentioned features were acquired from the single-trial ERPs and were averaged across the trials to generate the average ERP features to be used for classification.

### 2.6 Feature Selection

In order to acquire a set of optimal features that allows us to differentiate the three prime affect types, we employed a dimensionality reduction technique called linear local Fisher discriminant analysis (LFDA) [53]. LFDA transforms the high-dimensional data samples into a low-dimensional space while most of the intrinsic information is preserved [53]. This technique combines the ideas of Fisher discriminant analysis (FDA) and locality preserving projection (LPP). As a result, the between-class separability is increased, and within-class local structure is preserved. The samples ($x_{i}\in R^{n}$) in *n*-dimensional space are transformed to an *r*-dimensional space (we set *r* = 5) by using an *n* × *r* transformation matrix **T** as follows:

$$\begin{array}{c} z_{i}=T^{⊺}x_{i}, \end{array}$$ (2)

where $z_{i}\in R^{r}\left(1\leq r\leq n\right)$ are the samples in the reduced space (embedded samples).

The features are normalized for each participant by using Z-scores, where the mean is set to zero and the variance is set to 1. The high-dimensional normalized ERP feature set is fed to the LFDA, and the resultant embedded samples are provided to the classifiers.

For each classifier, optimized feature selection through LFDA was carried out.

## 3 Learning Algorithms: An Overview

We applied two different classification algorithms to infer the prime affect from the ERP data: SVMs and AdaBoost classifiers. These algorithms have successfully been applied to various classification problems [54–57]. A brief review of the theory behind the two learning algorithms is given in the following subsections.

### 3.1 Support Vector machines

Support vector machines (SVMs), introduced by Vapnik [58], are large margin classifiers. In the context of decoding information from EEGs, SVMs have exhibited satisfactory classification rates [59, 60]. In addition, they are known to have good generalization performance (i.e., error rate on test sets), and insensitivity to overtraining and to the curse-of-dimensionality.

Let us consider a training set of *m* vectors $x_{i}\in R^{n}$, where *x*_*i*_ belongs to an *n*-dimensional feature space X. Each vector *x*_*i*_ is associated with a label *y*_*i*_, where *y*_*i*_ belongs to a finite label space Y. For binary classification, we assume Y={-1,+1}, i.e., the prime affect type ({positive, negative} or {positive, neutral} or {negative, neutral}). Let us consider a hyperplane *w* ⋅ *x* + *b* = 0, where *w* is the normal to the plane, ‖*w*‖ is the Euclidean norm of *w*, and |*b*|/‖*w*‖ is the perpendicular distance from the hyperplane to the origin. Also, assume that the hyperplane separates the two classes in some space H and no prior knowledge is available about the data distribution. Then, the optimal hyperplane is the one that maximizes the margin. The optimal values of *w* and *b* are obtained by solving the constrained minimization problem using Lagrange multipliers *α* = *α*_1_, *α*_2_, …, *α*_*m*_:

$$\begin{array}{c} f\left(x\right)=\mathrm{sign}\left(\sum_{i=1}^{m}\alpha_{i}y_{i}K\left(x_{i},x\right)+b\right), \end{array}$$ (3)

where *K*(*x*_*i*_, *x*) is the kernel function. We refer the readers to [58] for more details on SVMs. The multi-class problem is formulated using the ‘one-against-all’ (OAA) strategy which constructs *k* (class labels) binary SVM classifiers, each of which distinguishes one class from the rest. The OAA strategy seems to be robust for cases having a small number of classes and a small set of training samples.

We trained the SVMs using the radial basis function (RBF) (a.k.a. Gaussian) kernel. It is highly effective in problems where the relationship between the class labels and the attributes is non-linear. The optimal values of the parameters such as RBF width *σ* and the regularization constant *C* are set by cross-validation. This results in the values *σ* = 8 and *C* = 1 for average ERP features, and *σ* = 2 and *C* = 1 for single-trial ERP features.

### 3.2 AdaBoost

The AdaBoost algorithm proposed by Freund and Schapire has been successfully applied in numerous classification problems [61–65]. It is a type of learning algorithm that combines many simple and moderately inaccurate classifiers into a single highly accurate classifier.

The AdaBoost algorithm repeatedly calls a given weak learning algorithm in a series of iterations *t* = 1, 2, …, *T*. The weak learner accepts the sample set *S* = {(*x*_1_, *y*_1_), (*x*_2_, *y*_2_), …, (*x*_*m*_, *y*_*m*_)} along with a distribution *D*_*t*_ over {1, 2, …, *m*} and outputs a weak hypothesis $h_{t}:X\to \left\{-1,+1\right\}$. *D*_*t*_ denotes the distribution or a set of weights for the training set. Initially, all weights are set equally and are updated in the subsequent iterations in such a way that the misclassified samples assume higher weights and the correctly classified samples the lower weights. This technique forces the weak learner in the subsequent round to focus on the hard sample [61, 62]. For each instance *x*, the sign of *h*_*t*_(*x*) identifies the predicted class label, and the absolute value gives the confidence in this classification.

The final hypothesis *H* is computed as a weighted majority vote of *T* weak hypotheses *h*_*t*_ with *α*_*t*_ being the weight assigned to *h*_*t*_. Therefore:

$$\begin{array}{c} H\left(x\right)=\mathrm{sign}\sum_{t=1}^{T}\alpha_{t}h_{t}\left(x\right). \end{array}$$ (4)

The AdaBoost is *adaptive* in that it adapts to the error rate of individual weak hypotheses. The AdaBoost algorithm has been extended to handle multi-class case where the goal is to find weak hypotheses with small pseudo-loss rather than hypotheses whose classification error is small. This is often referred to as AdaBoost.M2 [61].

For a given training sample (*x*_*i*_, *y*_*i*_), where $x_{i}\in X$, and $y_{i}\in Y=\left\{1,2,..,k\right\}$, the hypothesis *h* is used to answer *k* − 1 binary questions where *k* is the number of distinct class labels (*k* = 3). For an instance *x*_*i*_ and incorrect label *y* ≠ *y*_*i*_, assume a weight *q*(*i*, *y*) associated with the question that discriminates *y* from the correct label *y*_*i*_. Provided with *D*_*t*_ and label weighting function *q*_*t*_, the pseudo-loss of *h*_*t*_ is expressed as:

$$\begin{array}{c} \epsilon_{t}=\frac{1}{2}\sum_{i=1}^{m}D_{t}\left(i\right)\left(1-h_{t}\left(x_{i},y_{i}\right)+\sum_{y\neq y_{i}}q_{t}\left(i,y\right)h_{t}\left(x_{i},y\right)\right). \end{array}$$

We performed several classification tests with a decision-tree based AdaBoost algorithm with *T* = 10, 20, 30, 40, and 50. The value *T* = 20 yielded a good compromise between the computation time and classification accuracy, and is chosen for binary as well as multi-class problems. In comparison with SVM, no parameter tuning (except *T*) is required for AdaBoost. Further, SVMs are more computationally demanding than AdaBoost because SVM requires quadratic programming, whereas AdaBoost requires only linear programming.

## 4 Results and Discussion

In this section, we present the results for:

- Behavioral data (Likert scores) analysis for the experiment with and without subliminal primes
- EEG data analysis for the experiment with subliminal primes
- Decoding of the ERP data using the learning algorithms:
  - SVM (average-trial and single-trial classification)
  - AdaBoost (average-trial and single-trial classification)

### 4.1 Analysis of behavioral data

The responses on a 7-point Likert scale for each participant in the experiment with (40 subjects) and without (10 subjects) subliminal primes were averaged across the trials within each affect condition (positive, negative, and neutral) and then analyzed by means of one-way repeated measures ANOVA and two-tailed paired t-tests to determine the effect of subliminal priming on participant’s likability ratings on images.

The repeated measures ANOVA test with three conditions was significant for the experiment with subliminal primes (p = 1.11E-16<0.05), indicating significant differences in the Likert scores across the three affect conditions. However, no such effect was observed in the experiment without subliminal primes (p = 0.861).

The priming effect on behavior was further examined with the help of a two-tailed paired t-test for each pair of conditions (see Table 1). For the experiment with primes, the test returned significant results for positive-negative (p = 1.41E-09) and positive-neutral (p = 3.36E-12) pairs, implying a strong bias in the likeness judgment toward positive for the images in the positively primed condition compared to that of the negative condition. The effect of negative primes on behavior was, however, not evident in the data (p = 0.596 for negative-neutral). A possible explanation for this might be the weak association between the negative prime words and the stimuli images compared to that of the positive. The mean Likert score ratings for positive, negative, and neutral conditions were 5.02 (SD = 0.46), 4.48 (SD = 0.43), and 4.51 (SD = 0.34), respectively in the experiment with primes. Conversely, all three t-tests were insignificant for the experiment without primes (see Table 1). The mean Likert score ratings in this experiment were 4.67 (SD = 0.48), 4.61 (SD = 0.40), and 4.57 (SD = 0.62) for positive, negative, and neutral conditions, respectively.

**Table 1:** The p-values obtained from paired-samples t-test performed over the average response scores corresponding to positive-negative (Pos-Neg), positive-neutral (Pos-Neu), and negative-neutral (Neg-Neu) pairs.

Paired-samples t-test		
Prime pair	Experiment with primes	Experiment without primes
Pos-Neg	1.41E-09*	0.709
Pos-Neu	3.36E-12*	0.692
Neg-Neu	0.596	0.758

* Significant at p<0.05

In summary, the differences among positive, negative, and neutral conditions were observed only in the experiment with subliminal primes and not in the one without primes. This confirms that the observed differences in behavior are purely due to the effect of subliminal primes shown prior to the stimuli images and not due to the physical and subjective qualities of the supraliminal images.

### 4.2 Analysis of EEG data

**Fig 3:**
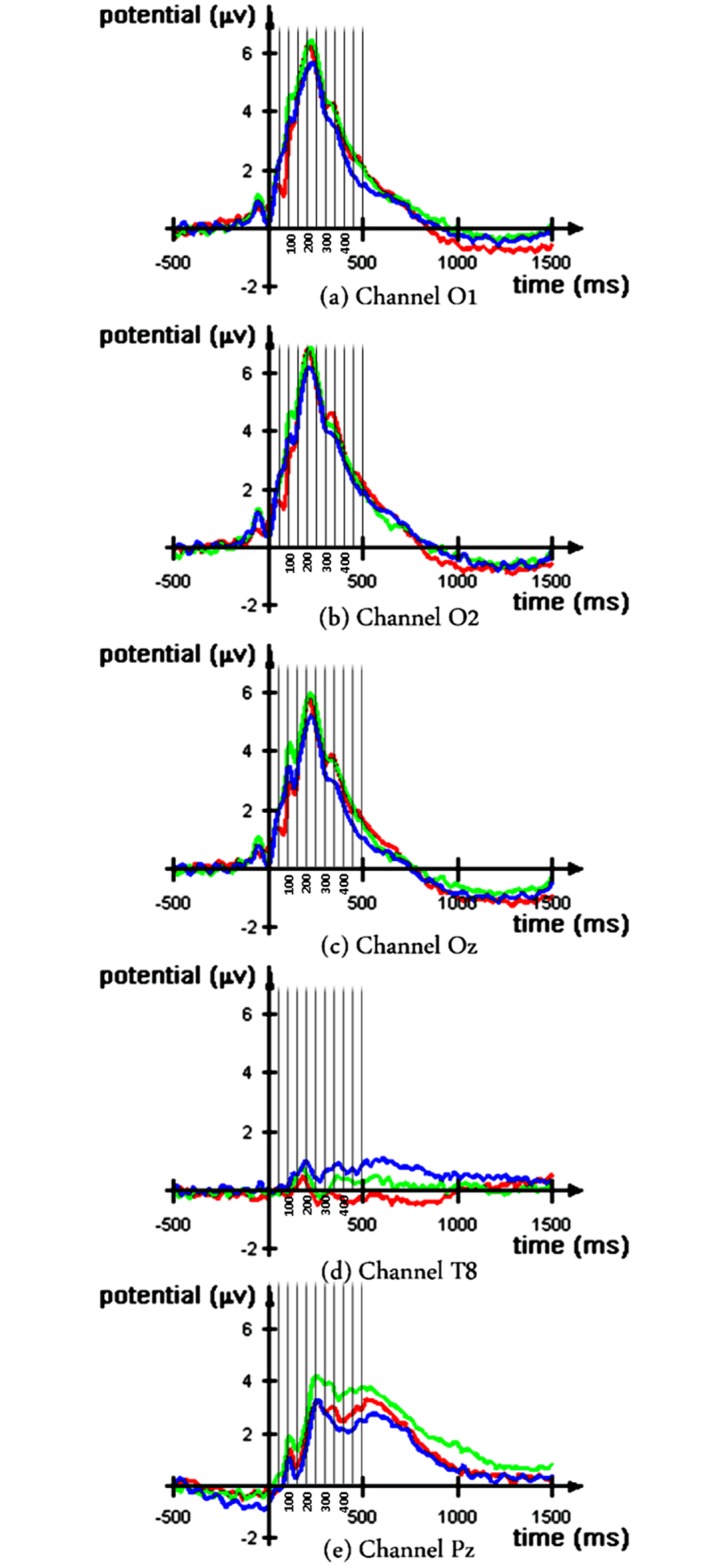
Grand ERP average for positive (in red), negative (in green), and neutral (in blue) prime affect types.

The grand ERP averages at different channels reflect the differences in brain activity among positive, negative, and neutral conditions at the early (50–100 ms) and late (400–450 ms) latencies (see Fig 3). The modulations in the EEG signal before the image onset could be due to the effect of prime word and the use of filter. We observed the N400 effect at channel Pz, associated with lexical priming [66]. One-way repeated measures ANOVA tests were carried out to examine the difference in brain activity among the conditions. The artifact-free ERP signals corresponding to three different conditions were first averaged across the trials, and then the mean amplitudes from discrete time windows were extracted using a window of length 25 ms for each subject. The mean amplitudes at discrete time windows corresponding to different prime affect conditions were analyzed by means of ANOVA tests. The p-values are summarized in Tables 2 and 3. It is interesting to note that the ANOVA test showed a significant difference between the negative and the neutral conditions in 400–425 ms and 425–450 ms time windows of channel Pz. Thus, the effect of negative primes, which was not visible in the behavioral data, was observed and confirmed through ERP analysis. This finding emphasizes the relevance of ERP studies in detecting a subliminal priming effect, which is rather subtle.

**Table 2:** The one-way repeated measures ANOVA test results for the windowed average ERPs with 25 ms analysis window that yield lowest p-values.

	50–75 ms			75–100 ms			300–325 ms		
Ch	P-Ng	P-Nu	N-Nu	P-Ng	P-Nu	N-Nu	P-Ng	P-Nu	N-Nu
O1	5.1E-03*	6.2E-03*	0.90	1.4E-05*	1.5E-04*	0.51	0.51	0.52	0.18
O2	3.8E-03*	7.9E-03*	0.57	4.2E-06*	3.9E-04*	0.45	0.87	0.40	0.50
Oz	3.7E-03*	0.02*	0.89	6.6E-06*	5.8E-04*	0.38	0.67	0.27	0.15
T8	0.98	0.48	0.38	0.47	0.38	0.80	0.83	0.06	0.05
Pz	0.12	0.94	0.10	0.11	0.91	0.15	0.07	0.82	0.02*

* Significant at p<0.05

**Table 3:** The one-way repeated measures ANOVA test results for the windowed average ERPs with 25 ms analysis window that yield lowest p-values.

	350–375 ms			400–425 ms			425–450 ms		
Ch	P-Ng	P-Nu	N-Nu	P-Ng	P-Nu	N-Nu	P-Ng	P-Nu	N-Nu
O1	0.84	0.16	0.17	0.60	0.28	0.09	0.57	0.23	0.08
O2	0.38	0.15	0.52	0.97	0.62	0.58	0.62	0.78	0.44
Oz	0.87	0.10	0.13	0.55	0.32	0.12	0.46	0.35	0.10
T8	0.06	0.01*	0.22	0.01*	4.9E-03*	0.32	7.7E-03*	7.8E-03*	0.45
Pz	0.20	0.24	7.7E-03*	0.06	0.50	6.7E-03*	0.05	0.28	3.2E-03*

* Significant at p<0.05

The effect was prominent in the occipital, lower temporal, and parietal lobes, and the difference was mainly between positive-negative, and positive-neutral pairs. The consistent differences among the three affect types demonstrated an overall priming effect.

The role of left/right dorsolateral prefrontal cortex (DLPFC) in predicting the neural activity of fMRI associated with sentence polarities was address in [67]. It was claimed that the right hemisphere (RDLPFC) can predict the sentence polarity with highest accuracy as compared to left hemisphere (LDLPFC). As can be seen from Table 2, the highest significant p-value was reported at channel O2, which is located at the right hemisphere. This is in line with the statement in [67].

### 4.3 Decoding ERPs

Here, we discuss the performance of the applied classifiers (SVM and AdaBoost) in inferring the prime affect type from the ERPs. We focus on two major classification tasks: (i) average-ERP classification and (ii) single-trial ERP classification.

#### 4.3.1 Average-trial ERP classification

**Performance evaluation:** Leave-one-subject-out cross-validation (LOSO-CV) was adopted to assess the performance of the classifiers in the average-trial ERP classification. The training set comprised the feature set of 39 participants’ average-trial ERP data. The model was then tested against the remaining subject. The process was repeated until all the subjects were employed as a test set. Finally, we report confusion matrices and measure the classifier accuracy, which is the average accuracy of all the subjects. The confusion matrices for a multi-class and a binary-class (positive-negative) classifiers are given in Tables 4 and 5, respectively; the confusion matrices for positive-neutral and negative-neutral classifiers are constructed similarly.

**Table 4:** Confusion matrix for a multiclass classifier.

		predicted		
		pos	neg	neu
actual	pos	x	x	x
	neg	x	x	x
	neu	x	x	x

**Table 5:** Confusion matrix for a positive-negative binary classifier.

		predicted	
		pos	neg
actual	pos	x	x
	neg	x	x

**Results:** Classification was performed using the average ERP features (averaged across the trials for each prime affect type) acquired from the 0–500 ms segment of the ERP, after the stimulus onset.

For each binary SVM classifier, the classification rate (% accuracy) for LOSO-CV is presented in Table 6. The highest classification accuracies obtained for SVMs were 95.0%, 87.5%, and 85.0% for positive-negative, positive-neutral, and negative-neutral, respectively. The features from the channels located at the central, temporal, and parietal lobes were found to be significant for discerning negative from positive, and also from neutral. However, features from only temporal and parietal lobes were required to discriminate between positive and neutral samples.

**Table 6:** Binary SVM classifier performance for average-ERP data.

SVM	Accuracy (%)	Confusion Matrix (%)	Input Features
Pos-Neg	95.0	$\left[\begin{array}{cc} 97.5 & 2.5\\ 7.5 & 92.5 \end{array}\right]$	relative power at channel T7, DWT coefficients (sym8) at channel P4, amplitude at channel C4, and PSD values at channel Pz.
Pos-Neu	87.5	$\left[\begin{array}{cc} 85.0 & 15.0\\ 10.0 & 90.0 \end{array}\right]$	relative power at channel T8, DWT coefficients (db4) at channel P7, amplitude at channel T7, and PSD values at channel T7.
Neg-Neu	85.0	$\left[\begin{array}{cc} 80.0 & 20.0\\ 10.0 & 90.0 \end{array}\right]$	relative power at channel P4, DWT coefficients (sym8) at channel C4, amplitude at channel P4, and PSD values at channel T8.

To further investigate and confirm that the participant’s mental state could easily be inferred from the average ERP features with a high performance rate, we conducted similar classification tasks using AdaBoost classifier. Maximum classification rates of 91.25%, 92.50%, and 81.25% were attained with AdaBoost for positive-negative, positive-neutral, and negative-neutral, respectively (see Table 7). The highest individual classification performance was accomplished when using ERP data from channels at locations other than frontal. We did not notice any differences in decoding performance when training with features from the right and left hemispheres in any individual classifiers.

**Table 7:** Binary AdaBoost classifier performance for average-ERP data.

AdaBoost	Accuracy (%)	Confusion Matrix (%)	Input Features
Pos-Neg	91.25	$\left[\begin{array}{cc} 95.0 & 5.0\\ 12.5 & 87.5 \end{array}\right]$	relative power at channel C4, DWT coefficients (db2) at channel C4, amplitude at channel O1, and PSD values at channel P8.
Pos-Neu	92.50	$\left[\begin{array}{cc} 92.5 & 7.5\\ 7.5 & 92.5 \end{array}\right]$	relative power at channel T7, DWT coefficients (db2) at channel O1, amplitude at channel O1, and PSD values at channel P8.
Neg-Neu	81.25	$\left[\begin{array}{cc} 85.0 & 15.0\\ 22.5 & 77.5 \end{array}\right]$	relative power at channel T7, DWT coefficients (db2) at channel Oz, amplitude at channel Pz, and PSD values at channel P8.

The performance of the individual binary classifiers of SVM and AdaBoost was slightly different, but both were found to be effective for the classification problem at hand. This decoding analysis revealed that different prime affect types induce significant changes in the ERP waveforms, which can be identified by means of any powerful classifier with appropriately tuned parameters and optimally selected input features. In other words, it is possible to reliably decode one’s mental states, induced by subliminal primes, using ERPs.

In addition, we investigated multi-class classification problems using the average ERP data. Satisfactory performance results of 70% and 61.67% accuracy rates were obtained for SVM and AdaBoost, respectively. The sensitivity of positive, negative, and neutral classes was 80.00%, 72.50%, and 57.50%, respectively for SVM. The figures were in the order of 67.50%, 75.00%, and 42.50% for AdaBoost (see Table 8). In summary, the SVM multi-class classifier outperformed AdaBoost.

**Table 8:** Performance of the multiclass classifiers for average ERP data.

Classifier	Accuracy (%)	Confusion Matrix (%)	Input Features
SVM	70.00	$\left[\begin{array}{ccc} 80.0 & 5.0 & 15.0\\ 15.0 & 72.5 & 12.5\\ 15.0 & 27.5 & 57.5 \end{array}\right]$	relative power at channel C4, DWT coefficients (db4) at channel P8, amplitude at channel P7, and PSD values at channel F7.
AdaBoost	61.67	$\left[\begin{array}{ccc} 67.5 & 30.0 & 2.5\\ 15.0 & 75.0 & 10.0\\ 17.5 & 40.0 & 42.5 \end{array}\right]$	relative power at channel O1, DWT coefficients (sym8) at channel P7, amplitude at channel P4, and PSD values at channel T8.

**Fig 4:**
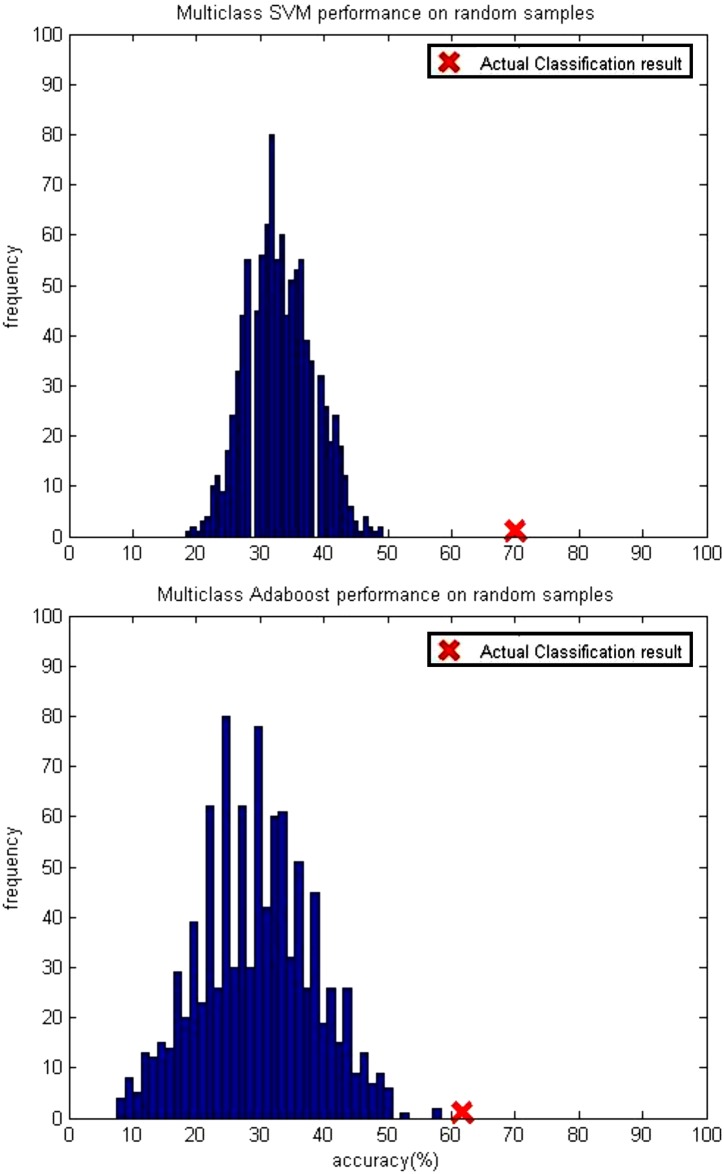
Multiclass SVM and AdaBoost performance on randomly permuted data.

The multiclass SVM and AdaBoost classifier performance were statistically validated by performing identical classification procedures on randomly permuted data (see Fig 4). Thousand synthetic data sets were generated by randomly assigning the class labels to the data. The performance on the actual set is marked using ‘*X*’. For both SVM and AdaBoost, the performance on the synthetic data set was not better than the one on the actual set. As can be seen from Fig 4, the highest accuracy obtained with SVM and AdaBoost were 49.17% and 58.33% respectively.

#### 4.3.2 Single-trial ERP classification

**Performance evaluation:** We applied leave-one-subject-out cross-validation (LOSO-CV) and leave-one-trial-out cross-validation (LOTO-CV) to assess subject-independent and subject-dependent models of single-trial classification, respectively. In the subject-independent approach, a classifier was generated from the training set, comprising of the feature set of 39 participants’ single-trial ERPs, and the resulting classifier was tested against the remaining subject’s single-trial ERPs. The process was repeated for all subjects. Finally, we calculated the classifier accuracy, i.e., the average accuracy of all the subjects. On the other hand, the subject-dependent model reserved one trial for testing purposes and used the remaining trials (approximately 99 trials in the case of the binary classifier) for training. Here, the final classifier accuracy was the average accuracy of all the trials of the 40 subjects.

**Results:** To make use of the information available in all single-trial ERPs, we trained classifiers with single-trial ERP features. Both SVM and AdaBoost performances were examined. Subject-independent and subject-dependent approaches were carried out. Tables 9 and 10 show the performance of subject-independent and subject-dependent classifiers respectively when using features from single-trial ERPs. For the subject-independent case, both SVM and AdaBoost performance were similar in terms of accuracies: 59.42%, 58.49%, and 53.67%, respectively, for positive-negative, positive-neutral, and negative-neutral SVM classifiers, and 59.80%, 58.20%, and 54.00%, respectively, for positive-negative, positive-neutral, and negative-neutral AdaBoost classifiers. As expected, for the subject-dependent case, the prediction accuracies were higher: 65.03%, 65.16%, and 62.65% for positive-negative, positive-neutral, and negative-neutral SVM classifiers, respectively, and 67.65%, 67.34%, and 63.23% for positive-negative, positive-neutral, and negative-neutral, respectively, for the AdaBoost classifiers.

**Table 9:** Performance of subject-independent classifiers for single-trial ERP data.

Classifier		Accuracy (%)	Input Features
SVM subject-independent	Pos-Neg	59.42	relative power at channel C4, DWT coefficients (db4) at channel O1, amplitude at channel P8, and PSD values at channel T7.
	Pos-Neu	58.49	relative power at channel O1, DWT coefficients (db4) at channel O1, amplitude at channel P7, and PSD values at channel C4.
	Neg-Neu	53.67	relative power at channel P4, DWT coefficients (db4) at channel O1, amplitude at channel O1, and PSD values at channel P8.
AdaBoost subject-independent	Pos-Neg	59.80	relative power at channel P4, DWT coefficients (db4) at channel P8, amplitude at channel O1, and PSD values at channel C4.
	Pos-Neu	58.20	relative power at channel C4, DWT coefficients (db4) at channel P4, amplitude at channel P7, and PSD values at channel Pz.
	Neg-Neu	54.00	relative power at channel F7, DWT coefficients (db4) at channel O1, amplitude at channel Oz, and PSD values at channel P8.

**Table 10:** Performance of the subject-dependent classifiers for single-trial ERP data.

Classifier		Accuracy (%)	Input Features
SVM subject-dependent	Pos-Neg	65.03	relative power at channel F7, DWT coefficients (db4) at channel O1, amplitude at channel T8, and PSD values at channel O1.
	Pos-Neu	65.16	relative power at channel C4, DWT coefficients (db4) at channel Oz, amplitude at channel O1, and PSD values at channel T7.
	Neg-Neu	62.65	relative power at channel O1, DWT coefficients (db4) at channel P7, amplitude at channel T7, and PSD values at channel O2.
AdaBoost subject-dependent	Pos-Neg	67.65	relative power at channel O1, DWT coefficients (db4) at channel P7, amplitude at channel F7, and PSD values at channel T7.
	Pos-Neu	67.34	relative power at channel O2, DWT coefficients (db4) at channel P8, amplitude at channel F7, and PSD values at channel O2.
	Neg-Neu	63.23	relative power at channel F7, DWT coefficients (db4) at channel T7, amplitude at channel T8, and PSD values at channel T8.

**Fig 5:**
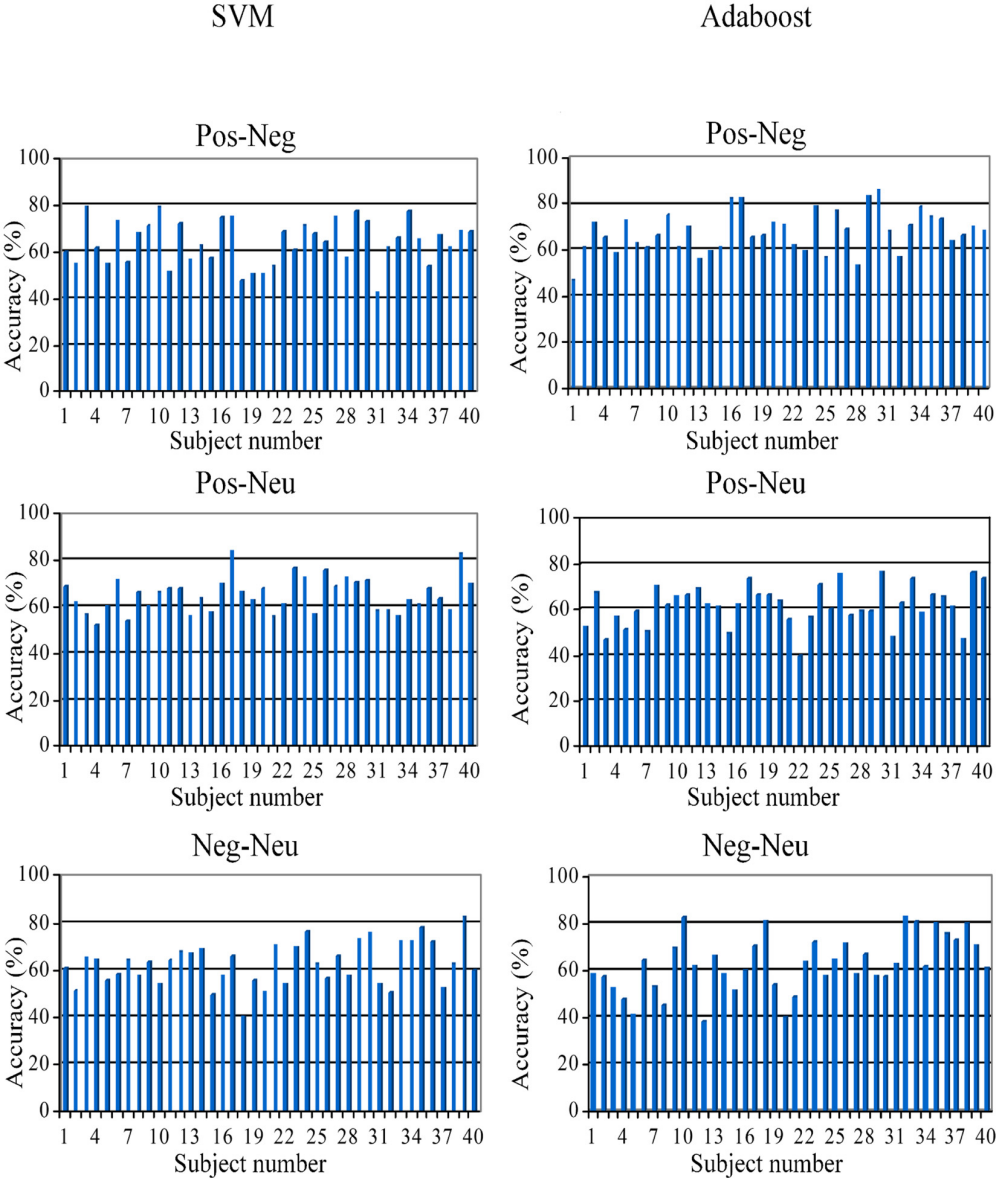
Single-trial ERP classification results (SVM (left) and AdaBoost (right)) of individual subjects (subject#1 to subject#40).

Fig 5 shows single-trial SVM and AdaBoost classification results for individual subjects. For some subjects, classification rates of 85% were achieved for single-trial ERPs. This finding further emphasizes that the ERPs encode information about the different kinds of primes.

#### 4.3.3 SVM and AdaBoost performance on identical input feature sets

The best performance results of SVM and AdaBoost classifiers on average-trial and single-trial ERP features were given in the previous sections. It is also interesting to compare the results on identical input feature sets as shown in Tables 11–14. The features that yielded best classification performance for binary SVM did not perform well for AdaBoost, and vice versa (see Tables 11 and 12). This is due to the fact that the features were optimized for each classifier separately. Subject independent and subject dependent performance on identical input features were computed and submitted in Tables 13 and 14.

**Table 11:** Binary SVM and AdaBoost classifier performance for average-ERP data using identical input features.

Classifier	Accuracy (%)		Input Features
	SVM	AdaBoost	
Pos-Neg	61.25	91.25	relative power at channel C4, DWT coefficients (db2) at channel C4, amplitude at channel O1, and PSD values at channel P8.
Pos-Neu	52.50	92.50	relative power at channel T7, DWT coefficients (db2) at channel O1, amplitude at channel O1, and PSD values at channel P8.
Neg-Neu	61.25	81.25	relative power at channel T7, DWT coefficients (db2) at channel Oz, amplitude at channel Pz, and PSD values at channel P8.
Pos-Neg	95.0	70.00	relative power at channel T7, DWT coefficients (sym8) at channel P4, amplitude at channel C4, and PSD values at channel Pz.
Pos-Neu	87.5	62.50	relative power at channel T8, DWT coefficients (db4) at channel P7, amplitude at channel T7, and PSD values at channel T7.
Neg-Neu	85.0	53.75	relative power at channel P4, DWT coefficients (sym8) at channel C4, amplitude at channel P4, and PSD values at channel T8.

**Table 12:** Performance of the multiclass classifiers for average ERP data using identical input features.

Accuracy (%)		Input Features
SVM	Adaboost	
70.00	44.17	relative power at channel C4, DWT coefficients (db4) at channel P8, amplitude at channel P7, and PSD values at channel F7.
40	61.67	relative power at channel O1, DWT coefficients (sym8) at channel P7, amplitude at channel P4, and PSD values at channel T8.

**Table 13:** Single-trial subject dependent and subject independent SVM classifier performance on identical input features.

Classifier	Accuracy (%)		Input Features
	Subject independent	Subject dependent	
Pos-Neg	59.52	62.20	relative power at channel C4, DWT coefficients (db4) at channel O1, amplitude at channel P8, and PSD values at channel T7.
Pos-Neu	58.49	61.39	relative power at channel O1, DWT coefficients (db4) at channel O1, amplitude at channel P7, and PSD values at channel C4.
Neg-Neu	53.67	55.83	relative power at channel P4, DWT coefficients (db4) at channel O1, amplitude at channel O1, and PSD values at channel P8.
Pos-Neg	54.07	65.03	relative power at channel F7, DWT coefficients (db4) at channel O1, amplitude at channel T8, and PSD values at channel O1.
Pos-Neu	55.59	65.16	relative power at channel C4, DWT coefficients (db4) at channel Oz, amplitude at channel O1, and PSD values at channel T7.
Neg-Neu	50.60	62.65	relative power at channel O1, DWT coefficients (db4) at channel P7, amplitude at channel T7, and PSD values at channel O2.

**Table 14:** Single-trial subject dependent and subject independent AdaBoost classifier performance on identical input features.

Classifier	Accuracy (%)		Input Features
	Subject independent	Subject dependent	
Pos-Neg	59.80	62.25	relative power at channel P4, DWT coefficients (db4) at channel P8, amplitude at channel O1, and PSD values at channel C4.
Pos-Neu	58.20	61.75	relative power at channel C4, DWT coefficients (db4) at channel P4, amplitude at channel P7, and PSD values at channel Pz.
Neg-Neu	54.00	58.06	relative power at channel F7, DWT coefficients (db4) at channel O1, amplitude at channel Oz, and PSD values at channel P8.
Pos-Neg	57.48	67.65	relative power at channel O1, DWT coefficients (db4) at channel P7, amplitude at channel F7, and PSD values at channel T7.
Pos-Neu	54.96	67.34	relative power at channel O2, DWT coefficients (db4) at channel P8, amplitude at channel F7, and PSD values at channel O2.
Neg-Neu	52.60	63.23	relative power at channel F7, DWT coefficients (db4) at channel T7, amplitude at channel T8, and PSD values at channel T8.

## 5 Conclusions

The current study investigated the changes in behavioral and electrophysiological responses to relatively natural and neutral images, after being subliminally exposed to three different types of prime words, which were deliberately designed to generate positive, negative, and neutral emotional associations with the images. Consistent with previous related studies on subliminal priming, the results showed the significant effect of priming on subjective judgment. The analysis of the behavioral data demonstrated a shift in the likeness judgment toward the positive for the images in the positive prime affect condition compared to that of the negative condition. The significance of negative priming on image rating was not visible in the behavioral responses data. A similar experiment conducted in the absence of subliminal prime words confirmed that the difference obtained in the behavioral data of the primed experiment was due to the influence of priming.

More interestingly, we were curious to examine how this behavioral shift affects the brain signals (EEG in particular), which could be considered a more objective measure to assess the priming effect. We observed an early and late response difference in the ERPs among the three prime affect types, mainly in the posterior region. These intriguing findings inspired us to explore further to what extent the ERPs encode information relevant to the priming effect. Promising correct classification rates of 95.00%, 87.50%, and 85.00% were reported for positive-negative, positive-neutral, and negative-neutral binary SVM classifiers, respectively, and 91.25%, 92.50%, and 81.25% for AdaBoost classifiers using average ERP data. The performance of the multi-class problem was lower than that of the binary problems (70.00% and 61.67% for SVM and AdaBoost respectively), as expected, since it is a more difficult classification problem. In addition, the decoding accuracies of the single-trial ERP classifications were also reasonable, with accuracies of 80%–85% for certain subjects.

In summary, our results not only support the previous literature on priming, but also highlight the significance of ERP studies for gaining better understanding the brain-behavior correlations. The promising results could benefit research in areas such as brain and cognition research, and health science, and rehabilitation research. In addition, the results could also be used for motivational research, for instance, for subliminally motivating/influencing the staff and students for better productivity and creativity. Further research will need to be carried out to explore the short-term and long-term effects of priming on subjective and objective judgments of images, as well as whether a gender-specific effect can be observed.

## References

[1] Newell BR, Shanks DR. Unconscious influences on decision making: A critical review. Behavioral and Brain Sciences. 2014;37(01):1–19. 10.1017/S0140525X12003214 24461214

[2] Fazio RH. On the automatic activation of associated evaluations: An overview. Cognition & Emotion. 2001;15(2):115–141.

[3] Houwer JD. How do people evaluate objects? A brief review. Social and Personality Psychology Compass. 2009;3(1):36–48. 10.1111/j.1751-9004.2008.00162.x

[4] Hermans D, Houwer JD, Eelen P. The affective priming effect: Automatic activation of evaluative information in memory. Cognition & Emotion. 1994;8(6):515–533. 10.1080/02699939408408957

[5] Klauer KC, Roßnagel C, Musch J. List-context effects in evaluative priming. Journal of Experimental Psychology: Learning, Memory, and Cognition. 1997;23(1):246 9028030 10.1037//0278-7393.23.1.246

[6] Comesaña M, Soares AP, Perea M, Piñeiro AP, Fraga I, Pinheiro A. ERP correlates of masked affective priming with emoticons. Computers in Human Behavior. 2013;29(3):588–595.

[7] Marcos JL, Redondo J. Facilitation and interference of the automatic information processing on a reaction time task to threat-relevant stimuli. Psicothema. 2005;17(2):332–337.

[8] Murphy ST, Zajonc RB. Affect, cognition, and awareness: affective priming with optimal and suboptimal stimulus exposures. Journal of personality and social psychology. 1993;64(5):723 10.1037/0022-3514.64.5.723 8505704

[9] Lu Y, Zhang WN, Hu W, Luo YJ. Understanding the subliminal affective priming effect of facial stimuli: an ERP study. Neuroscience letters. 2011;502(3):182–185. 21827830 10.1016/j.neulet.2011.07.040

[10] Rotteveel M, de Groot P, Geutskens A, Phaf RH. Stronger suboptimal than optimal affective priming? Emotion. 2001;1(4):348 10.1037/1528-3542.1.4.348 12901397

[11] Winkielman P, Berridge KC, Wilbarger JL. Unconscious affective reactions to masked happy versus angry faces influence consumption behavior and judgments of value. Personality and Social Psychology Bulletin. 2005;31(1):121–135. 10.1177/0146167204271309 15574667

[12] Draine SC, Greenwald AG. Replicable unconscious semantic priming. Journal of Experimental Psychology: General. 1998;127(3):286 10.1037/0096-3445.127.3.286 9742717

[13] Greenwald AG, Draine SC, Abrams RL. Three cognitive markers of unconscious semantic activation. Science. 1996;273(5282):1699–1702. 10.1126/science.273.5282.1699 8781230

[14] Greenwald AG, Klinger MR, Liu TJ. Unconscious processing of dichoptically masked words. Memory & Cognition. 1989;17(1):35–47. 10.3758/BF03199555 2913455

[15] Otten S, Wentura D. About the impact of automaticity in the Minimal Group Paradigm: Evidence from affective priming tasks. European Journal of Social Psychology. 1999;29(8):1049–1071. 10.1002/(SICI)1099-0992(199912)29:8<1049::AID-EJSP985>3.0.CO;2-Q

[16] Gibbons H. Evaluative priming from subliminal emotional words: insights from event-related potentials and individual differences related to anxiety. Consciousness and Cognition. 2009;18(2):383–400. 19328727 10.1016/j.concog.2009.02.007

[17] Zhang Q, Lawson A, Guo C, Jiang Y. Electrophysiological correlates of visual affective priming. Brain research bulletin. 2006;71(1):316–323. 17113962 10.1016/j.brainresbull.2006.09.023 PMC1783676

[18] Zhang Q, Li X, Gold BT, Jiang Y. Neural correlates of cross-domain affective priming. Brain research. 2010;1329:142–151. 10.1016/j.brainres.2010.03.021 20298681 PMC2857548

[19] Schacht A, Sommer W. Time course and task dependence of emotion effects in word processing. Cognitive, Affective, & Behavioral Neuroscience. 2009;9(1):28–43. 10.3758/CABN.9.1.28 19246325

[20] Junghöfer M, Bradley MM, Elbert TR, Lang PJ. Fleeting images: a new look at early emotion discrimination. Psychophysiology. 2001;38(02):175–178. 10.1111/1469-8986.3820175 11347862

[21] Schupp HT, Junghöfer M, Weike AI, Hamm AO. The selective processing of briefly presented affective pictures: An ERP analysis. Psychophysiology. 2004;41(3):441–449. 10.1111/j.1469-8986.2004.00174.x 15102130

[22] Cuthbert BN, Schupp HT, Bradley MM, Birbaumer N, Lang PJ. Brain potentials in affective picture processing: covariation with autonomic arousal and affective report. Biological psychology. 2000;52(2):95–111. 10699350 10.1016/s0301-0511(99)00044-7

[23] Schupp HT, Cuthbert BN, Bradley MM, Cacioppo JT, Ito T, Lang PJ. Affective picture processing: the late positive potential is modulated by motivational relevance. Psychophysiology. 2000;37(2):257–261. 10.1111/1469-8986.3720257 10731776

[24] Kotz SA, Paulmann S. Emotion, language, and the brain. Language and Linguistics Compass. 2011;5(3):108–125. 10.1111/j.1749-818X.2010.00267.x

[25] Daliri MR, Taghizadeh M, Niksirat KS. EEG Signature of Object Categorization from Event-Related Potentials. Journal of medical signals and sensors. 2013;3(1):37 24083136 PMC3785069

[26] Esghaei M, Daliri MR. Decoding of visual attention from LFP signals of macaque MT. 2014. 10.1371/journal.pone.0100381 PMC4076262 24979704

[27] Seif Z, Daliri MR. Evaluation of local field potential signals in decoding of visual attention Cognitive Neurodynamics; p. 1–14. 10.1007/s11571-015-9336-2 PMC4568000 26379801

[28] Behroozi M, Daliri MR. Predicting brain states associated with object categories from fMRI data. Journal of integrative neuroscience. 2014;13(04):645–667. 10.1142/S0219635214500241 25352153

[29] Haynes JD, Rees G. Decoding mental states from brain activity in humans. Nature Reviews Neuroscience. 2006;7(7):523–534. 10.1038/nrn1931 16791142

[30] Das K, Giesbrecht B, Eckstein MP. Predicting variations of perceptual performance across individuals from neural activity using pattern classifiers. Neuroimage. 2010;51(4):1425–1437. 20302949 10.1016/j.neuroimage.2010.03.030

[31] Philiastides MG, Ratcliff R, Sajda P. Neural representation of task difficulty and decision making during perceptual categorization: a timing diagram. The Journal of Neuroscience. 2006;26(35):8965–8975. 10.1523/JNEUROSCI.1655-06.2006 16943552 PMC6675324

[32] Philiastides MG, Sajda P. Temporal characterization of the neural correlates of perceptual decision making in the human brain. Cerebral cortex. 2006;16(4):509–518. 10.1093/cercor/bhi130 16014865

[33] Bode S, Sewell DK, Lilburn S, Forte JD, Smith PL, Stahl J. Predicting perceptual decision biases from early brain activity. The Journal of Neuroscience. 2012;32(36):12488–12498. 10.1523/JNEUROSCI.1708-12.2012 22956839 PMC6621270

[34] Bernat E, Bunce S, Shevrin H. Event-related brain potentials differentiate positive and negative mood adjectives during both supraliminal and subliminal visual processing. International Journal of Psychophysiology. 2001;42(1):11–34. 11451477 10.1016/s0167-8760(01)00133-7

[35] Fischler I, Bradley M. Event-related potential studies of language and emotion: words, phrases, and task effects. Progress in brain research. 2006;156:185–203. 17015080 10.1016/S0079-6123(06)56009-1

[36] Frühholz S, Jellinghaus A, Herrmann M. Time course of implicit processing and explicit processing of emotional faces and emotional words. Biological psychology. 2011;87(2):265–274. 21440031 10.1016/j.biopsycho.2011.03.008

[37] Herbert C, Kissler J, Junghöfer M, Peyk P, Rockstroh B. Processing of emotional adjectives: Evidence from startle EMG and ERPs. Psychophysiology. 2006;43(2):197–206. 10.1111/j.1469-8986.2006.00385.x 16712590

[38] Bayer M, Sommer W, Schacht A. P1 and beyond: Functional separation of multiple emotion effects in word recognition. Psychophysiology. 2012;49(7):959–969. 10.1111/j.1469-8986.2012.01381.x 22594767

[39] Herbert C, Junghofer M, Kissler J. Event related potentials to emotional adjectives during reading. Psychophysiology. 2008;45(3):487–498. 10.1111/j.1469-8986.2007.00638.x 18221445

[40] Kiefer M. The N400 is modulated by unconsciously perceived masked words: Further evidence for an automatic spreading activation account of N400 priming effects. Cognitive Brain Research. 2002;13(1):27–39. 11867248 10.1016/s0926-6410(01)00085-4

[41] Ross ED, Monnot M. Neurology of affective prosody and its functional—anatomic organization in right hemisphere. Brain and language. 2008;104(1):51–74. 17537499 10.1016/j.bandl.2007.04.007

[42] Kumar P, Mahmood F, Mohan DM, Wong K, Agrawal A, Elgendi M, et al. On the effect of subliminal priming on subjective perception of images: A machine learning approach. In: Engineering in Medicine and Biology Society (EMBC), 2014 36th Annual International Conference of the IEEE. IEEE; 2014. p. 5438–5441. 10.1109/EMBC.2014.6944856 25571224

[43] Oldfield RC. The assessment and analysis of handedness: the Edinburgh inventory. Neuropsychologia. 1971;9(1):97–113. 5146491 10.1016/0028-3932(71)90067-4

[44] Aarts H, Custers R, Wegner DM. On the influence of personal authorship: Enhancing experienced agency by priming effect information. Consciousness and Cognition. 2005;14(3):439–58. 16091264 10.1016/j.concog.2004.11.001

[45] Mitchell J, Macrae C, Schooler J, Rowe A, Milne A. Directed remembering: Subliminal cues alter non-conscious memory strategies. Memory. 2002;10(5):381–388. 10.1080/09658210244000207 12396650

[46] Lowery BS, Eisenberger NI, Hardin CD, Sinclair S. Long-term effects of subliminal priming on academic performance. Basic and Applied Social Psychology. 2008;29(2):151–7. 10.1080/01973530701331718

[47] Jasper HH. The ten twenty electrode system of the international federation. Electroencephalography and clinical neurophysiology. 1958;10:371–375. 10590970

[48] Delorme A, Makeig S. EEGLAB: an open source toolbox for analysis of single-trial EEG dynamics including independent component analysis. Journal of neuroscience methods. 2004;134(1):9–21. 15102499 10.1016/j.jneumeth.2003.10.009

[49] Makeig S, Bell AJ, Jung TP, Sejnowski TJ, et al Independent component analysis of electroencephalographic data Advances in neural information processing systems. 1996; p. 145–151.

[50] Daubechies I. Orthonormal bases of compactly supported wavelets. Communications on pure and applied mathematics. 1988;41(7):909–996. 10.1002/cpa.3160410705

[51] Mallat S. A wavelet tour of signal processing. Academic press; 1999.

[52] Soman K, et al Insight into wavelets: From theory to practice. PHI Learning Pvt. Ltd; 2010.

[53] Sugiyama M. Dimensionality reduction of multimodal labeled data by local fisher discriminant analysis. The Journal of Machine Learning Research. 2007;8:1027–1061.

[54] Cheng WC, Jhan DM. Triaxial Accelerometer-Based Fall Detection Method Using a Self-Constructing Cascade-AdaBoost-SVM Classifier. IEEE journal of biomedical and health informatics. 2013;17(2):411–419. 10.1109/JBHI.2012.2237034 24235113

[55] Stewart AX, Nuthmann A, Sanguinetti G. Single-trial classification of EEG in a visual object task using ICA and machine learning. Journal of neuroscience methods. 2014;228:1–14. 24613798 10.1016/j.jneumeth.2014.02.014

[56] Taghizadeh-Sarabi M, Daliri MR, Niksirat KS. Decoding Objects of Basic Categories from Electroencephalographic Signals Using Wavelet Transform and Support Vector Machines Brain topography. 2014; p. 1–14. 10.1007/s10548-014-0371-9 24838816

[57] Liao K, Xiao R, Gonzalez J, Ding L. Decoding individual finger movements from one hand using human EEG signals. PloS one. 2014;9(1):e85192 10.1371/journal.pone.0085192 24416360 PMC3885680

[58] Vapnik VN. An overview of statistical learning theory. Neural Networks, IEEE Transactions on. 1999;10(5):988–999. 10.1109/72.788640 18252602

[59] McFarland DJ, Anderson CW, Muller K, Schlogl A, Krusienski DJ. BCI meeting 2005-workshop on BCI signal processing: feature extraction and translation. IEEE transactions on neural systems and rehabilitation engineering. 2006;14(2):135 10.1109/TNSRE.2006.875637 16792278

[60] Lotte F, Congedo M, Lécuyer A, Lamarche F, Arnaldi B, et al A review of classification algorithms for EEG-based brain—computer interfaces. Journal of neural engineering. 2007;4 10.1088/1741-2560/4/2/R01 17409472

[61] Freund Y, Schapire R, Abe N. A short introduction to boosting. Journal-Japanese Society For Artificial Intelligence. 1999;14(771–780):1612.

[62] Schapire RE, Singer Y. Improved boosting algorithms using confidence-rated predictions. Machine learning. 1999;37(3):297–336. 10.1023/A:1007614523901

[63] Cristinacce D, Cootes TF. Facial feature detection using AdaBoost with shape constraints In: BMVC; 2003 p. 1–10.

[64] Lv F, Nevatia R. Recognition and segmentation of 3-d human action using hmm and multi-class adaboost In: Computer Vision—ECCV 2006. Springer; 2006 p. 359–372.

[65] Niu B, Cai YD, Lu WC, Li GZ, Chou KC. Predicting protein structural class with AdaBoost learner. Protein and peptide letters. 2006;13(5):489–492. 10.2174/092986606776819619 16800803

[66] Morris J, Porter JH, Grainger J, Holcomb PJ. Effects of lexical status and morphological complexity in masked priming: an ERP study. Language and cognitive processes. 2011;26(4–6):558–599. 10.1080/01690965.2010.495482 PMC3998725 24771954

[67] Behroozi M, Daliri MR. RDLPFC area of the brain encodes sentence polarity: a study using fMRI. Brain imaging and behavior. 2014;9(2):178–189. 10.1007/s11682-014-9294-z 24573772

